# Monkeypox in Syria: Highlighting an awareness issue

**DOI:** 10.1016/j.ijregi.2023.04.008

**Published:** 2023-04-20

**Authors:** Sarya Swed, Hidar Alibrahim, Haidara Bohsas, Ahmed Aljabali, Mohammad Badr Almoshantaf, Bisher Sawaf, Sheikh Shoib, Muhammad Mainuddin Patwary, Ebraheem Albazee, Ka Yiu Lee, Amr Farwati, Mohammed Najdat Seijari, Wael Hafez, Amine Rakab

**Affiliations:** 1 Faculty of Medicine Aleppo University, Aleppo, Syria; 2 Jordan University of Science and Technology; 3 Department of Neurosurgery, Ibn Al-Nafees Hospital, Damascus, Syria; 4 Department of Internal Medicine, Hamad Medical Corporation, Doha, Qatar; 5 JLNM Hospital, Rainawari, Srinagar; 6 Directorate of Health Services, J&K, India; 7 Environment and Sustainability Research Initiative, Khulna, 9208, Bangladesh; 8 Environmental Science Discipline, Life Science School, Khulna University, Khulna, 9208, Bangladesh; 9 Kuwait Institute for Medical Specializations, Kuwait City, Kuwait; 10 Department of Health Sciences, Mid Sweden University, Sweden; 11 NMC Royal Hospital, 16th Street, Khalifa City, Abu Dhabi, UAE; 12 Medical Research Division, Department of Internal Medicine, The National Research Centre, Cairo, Egypt; 13 Assistant Professor of Clinical Medicine, Medicine, Weill Cornell Medical College, Qatar

**Keywords:** Monkeypox, Awareness, Syria, Healthcare workers, Cross sectional

## Abstract

**Background:**

The World Health Organization (WHO) verified 780 cases of monkeypox across 27 countries between 13 May 2022 and 2 June 2022. The aim of our study was to assess the level of awareness of human monkeypox virus among Syrian medical students, general practitioners, medical residents, and specialists.

**Methods:**

A cross-sectional online survey was performed in Syria between May 2 and September 8, 2022. The survey consisted of 53 questions within the following three categories: demographic information, work-related details, and monkeypox knowledge.

**Results:**

In total, 1257 Syrian healthcare workers and medical students were enrolled in our study. The animal host and incubation time for monkeypox were correctly identified by just 2.7% and 33.3% of responders, respectively. Sixty percent of the study sample thought that the symptoms of monkeypox and smallpox are identical. No statistically significant associations were found between predictor variables and knowledge regarding monkeypox (*p*-value > 0.05).

**Conclusion:**

Education and awareness regarding monkeypox vaccinations are of paramount importance. It is essential that clinical doctors are adequately aware of this disease, in order to avoid an uncontrolled situation, as experienced with COVID-19.

## Highlights

• Awareness of human monkeypox virus was assessed among medical students and healthcare workers in Syria.
• There is a lack of understanding of monkeypox virus in the country.

## Background

The monkeypox virus (MPXV) is a double-stranded DNA virus encased in a protein shell. It is most common in central and western Africa [1], [2]. The first human monkeypox case was discovered in the Democratic Republic of the Congo (DRC) in 1970, following an intensive attempt to eliminate smallpox, and the illness still exists today [2], [3].

Vague symptoms, starting with a fever, may appear before the onset of chills, headaches, lethargy, asthenia, lymph node swellings, back pain, and myalgia (muscle pain). Rashes of varying sizes appear anywhere from 1 day to 5 days after the onset of fever, starting on the face and spreading to the rest of the body, arms, legs, and feet. The rash develops in stages, starting with macules, papules, vesicles (fluid-filled blisters), and pustules, and ending with crusts and scabs that drop off after the condition has improved. Inflammation might also be present in the pharyngeal, conjunctival, and vaginal mucosa [4]. It was determined in 1987 that the only diagnostic sign differentiating monkeypox (MPX) from smallpox and chickenpox is significant lymphadenopathy (varicella) [5].

The illness was discovered in a medical professional treating one of the first two cases of monkeypox in the UK [6]. Between January and December 2019, 113 instances of MPXV were reported, of which 45 were confirmed by the Nigeria Center for Disease Control (NCDC), in nine states: Oyo, Bayelsa, Lagos, Delta, Rivers, Enugu, Akwa Ibom, Anambra, and Cross River [7].

The two most likely MPXV transfer methods are from animal to human and from human to human. Viruses may spread from animals to humans via close contact or by consuming animal hosts. Touching the blood, body fluids, or wounds of infected animals can thus spread zoonotic infections. Cross-transmission has been linked with skin lesions on sick people, contaminated environments, or respiratory tract droplets [8].

After electing to utilize a conventional monitoring system early in the epidemic, the NCDC opted to deploy a prior version of the Surveillance, Outbreak Response Management, and Analysis System (SORMAS), which had been successfully used in Nigeria in October 2017. Subsequently, the National Centers for Disease Control and Prevention stated that SORMAS should be used [9]. One of the monitoring system's essential functions is increasing the capacity of healthcare personnel to notice cases and improve patient care [10].

Between May 13 and June 2, 2022, 780 monkeypox cases from 27 Member States, spread across four non-endemic WHO regions, were reported to or validated by WHO. Most reports confirmed patients with travel histories including visits to Europe and North America as opposed to West or Central Africa, where monkeypox is prevalent [11].

According to observational studies that have demonstrated poor knowledge of human monkeypox among healthcare workers, general practitioners need adequate training before they can be employed in an endemic setting. Environmental issues need further research to determine the reservoir hosts, and there should be an emphasis on educational initiatives to help the most affected [12].

Our study aimed to evaluate medical students, general practitioners (GPs), medical residents, and specialists’ knowledge of the human monkeypox virus in Syria.

## Methods

### Study design and setting

A cross-sectional online study was conducted in Syria between May 2 and September 8, 2022. The participants included medical students, general practitioners, medical residents, and specialists from all Syrian governorates. The study aims, the researchers’ identities, the opportunity to withdraw from participation, and the importance the researchers placed on their confidentiality were identified on the first page of the survey.

The survey was developed using data from the World Health Organization (WHO), the American Centers for Disease Control and Prevention (CDC), and cross-sectional research conducted in Indonesia [12]; it was then translated into Arabic. Convenience and snowball strategies were used to collect information from respondents. Because of security concerns, a Google form questionnaire was created and delivered to responders through social media platforms such as Facebook, WhatsApp, and Telegram. Additionally, participants were allowed to share the survey's URL on social media. The sample size was calculated using a standardized program called ‘select-statistics.co.uk’.

The following criteria were used in a statistical power analysis to calculate the suitable sample size: a population percentage of 36.5% (the proportion of participants in the Indonesian research who had sufficient knowledge of monkeypox [12]), a margin of error of 0.05, and a confidence level of 95%, giving a recommended sample size of 355.

On the Google form website 1269 people were asked to participate in this survey; however, 12 respondents declined, reducing the final sample size to 1257.

### Measures

The survey comprised 53 questions divided into three sections. On the first page was a question about willingness to participate in the study.

#### Sociodemographic variables and work-related characteristics

Fourteen questions were contained in this section, covering the participant's age, gender, marital status, place of residence, chronic illnesses, country of origin, academic year (for medical students), and specialty for residents and specialists). Participants were also asked whether they were a specialist or general practitioner, how long they had been practicing, whether they had attended conferences domestically or overseas, and whether they had more than 5 years’ experience. Participants were also asked whether they had studied anything about monkeypox in their medical curriculum, if they had previously heard of monkeypox, and how long ago they first heard about it (months, weeks, days, etc.). Participants were asked how they first learned about this virus (e.g. internet, social media, television, medical lectures).

#### General knowledge about the monkeypox virus

This section had 13 questions regarding the monkeypox virus's basic characteristics, such as its animal host (squirrels, dogs, monkeys, etc.), incubation period (1 to 7 days, 7 to 14 days, 24 to 28 days, etc.), the age group most vulnerable to infection, and how the illness is spread (human to human, animal to human, etc.). This part also included questions regarding the disease's signs and symptoms, duration (1 week, 2 to 4 weeks, etc.), diagnostic procedures (PCR testing, clinical diagnosis, etc.), examination sample types (blood, urine, stool, etc.), potentially serious side-effects (skin rashes, respiratory problems, digestive issues, etc.), methods of therapy (supportive care, antibiotics, antivirals, etc.), and preventative strategies (vaccination, avoidance of exposure, etc.). One mark was awarded for the correct response, while an incorrect response or ‘don't know’ scored zero.

#### Knowledge about the monkeypox virus

This section comprised 21 questions designed to assess further knowledge about the monkeypox virus, including the prevalence of the virus in Asia, Western and Central Africa, and in Syria. The section also investigated whether respondents thought the illness was viral or bacterial, as well as the similarities between monkeypox and smallpox symptoms. In addition, it included questions concerning the symptoms of monkeypox (e.g. lymphadenopathy and skin rashes) and treatment options for this illness. Again, one mark was awarded for a correct answer, while an incorrect response or ‘don't know’ received a score of zero.

Thus, the final scale used to evaluate clinicians’ and medical students’ knowledge of monkeypox ranged from 0 to 34.

### Pilot study

The applicability and readability of this questionnaire were tested on 50 randomly chosen members of the general population. Modifications were then made based on participant feedback. Then, using 50 volunteers, a pilot test was conducted to determine the reliability of the questionnaire. Cronbach's alpha ratings for the areas varied from 0.712 to 0.861, showing that the instrument maintained a high level of internal consistency. The questionnaire was released after the pilot study was over.

### Ethical considerations

IRB approval was received from the Ethical Society for Scientific Research in Syria (IRB ref: SA784). Participants were given a URL to access the online survey on Google; the first page included a question confirming whether or not the participants were willing to complete the questionnaire. Before completing the questionnaire, participants were directed to the next page, which contained comprehensive information on the research. Participants were asked to complete the questionnaire within 5–12 minutes. All responses were saved in a protected online database.

### Statistical analysis

Statistical analysis of the data was carried out using the IBM SPSS package (v. 28.0; IBM Corporation, Armonk, NY, USA). A *p*-value of 0.05 or below was considered statistically significant. Categorical variables relating to sociodemographic characteristics of the parents were presented using descriptive statistics and frequencies. For the statistical analysis, levels of knowledge were categorized into good or poor, based on two modified Bloom's cutoff criteria: 80% and 70% of the total score (i.e. if a participant answered at least 27 or no more than 24, respectively, of the total 34 questions correctly). A univariate analysis using the Mann–Whitney U-test (for non-normal continuous variables), *t*-test (for normal distributions of continuous variables), and chi-squared test (for categorical variables) was performed to determine factors influencing the knowledge level of participants. Next, a multivariate logistic regression analysis was conducted for the variables with significance (*p* ≤ 0.05) in the univariate analysis, in order to evaluate the odds ratios of the factors determining the knowledge levels of participants.

## Results

### Demographic characteristics

Table 1 provides a summary of the respondents’ demographic characteristics. This study included 1257 healthcare practitioners from every governorate in Syria; 61.7% (*n* = 775) of the participants were female. The majority of participants were single (81.9%, *n* = 1030) and were under 30 years old (87.7%, *n* = 1102). Approximately 81.8% (*n* = 1028) resided in city areas. A significant proportion of participants (47.4%, *n* = 596) had a moderate economic status, with 40.8% (*n* = 513) reporting a good economic status. The majority of responders (87.9%, *n* = 1105) had no chronic disease history.

**Table 1:** Unadjusted and multivariable logistic regression analysis, showing predictors of knowledge (using a cut-off of 70%) of human monkeypox infection, among medical students and healthcare professionals in Syria (good vs poor) (*n* = 1257)

Variables	*n* (%)	Good knowledge, *n* (%)	Unadjusted		Multivariable	
			Crude OR (95% CI)	*p*-value	Adjusted OR (95% CI)	*p*-value
**Gender**						
Male	482 (38.3)	1 (0.2)	Ref.			
Female	775 (61.7)	2 (0.3)	1.245 (0.113–13.762)	0.858		
**Age (years)**						
30 or under	1102 (87.7)	3 (0.3)	Ref.			
Over 30	155 (12.3)	0 (0)	0.00 (0.000–)	0.995		
**Social status**						
Single	1030 (81.9)	3 (0.3)	Ref.			
Married	216 (17.2)	0 (0)	0.129 (0.008–2.090)	0.150		
Divorced	9 (0.7)	0 (0)	0.150 (0.009–2.429)	0.182		
Widower	2 (0.2)	0 (0)	0.00 (0.000–)	0.997		
**Residence**						
City	1028 (81.8)	3 (0.3)	Ref.		Ref.	
Country	229 (18.2)	0 (0)	0.003 (0.001–0.009)	< 0.001	3.084 (0.000–)	1.000
**Economic status**						
Low	78 (6.2)	1 (1.3)	Ref.		Ref.	
Moderate	596 (47.4)	1 (0.2)	0.002 (0.000–0.012)	< 0.001	0.00 (0.000–)	1.000
Good	513 (40.8)	1 (0.2)	0.002 (0.000–0.014)	< 0.001	0.00 (0.000–)	0.999
High	70 (5.6)	0 (0)	0.00 (0.000–)	0.996	–	–
**Chronic disease**						
No	1105 (87.9)	3 (0.3)	Ref.			
Yes	152 (12.1)	0 (0)	0.00 (0.000–)	0.995		
**Occupation**						
Medical student	675 (53.7)	3 (0.4)	Ref.			
General practitioner	110 (8.8)	0 (0)	0.00 (0.000–)	0.996		
Resident	319 (25.4)	0 (0)	0.00 (0.000–)	0.992		
Specialist	153 (12.2)	0 (0)	0.00 (0.000–)	0.995		
**Academic year (student)**						
First	61 (9)	0 (0)	Ref.			
Second	134 (19.8)	1 (0.7)	0.008 (0.001–0.054)	< 0.001	0.271906 (0.000–)	1.000
Third	72 (10.6)	1 (1.4)	0.014 (0.002–0.101)	< 0.001	1.225120 (0.000–)	1.000
Fourth	106 (15.6)	0 (0)	0.000 (0.000–)	0.996	–	–
Fifth	166 (24.5)	0 (0)	0.000 (0.000–)	0.995	–	–
Sixth	139 (20.5)	1 (0.7)	0.007 (0.001–0.052)	< 0.001	0.999 (10.888–)	1.000
**Medical specialty**						
Anesthesia and resuscitation	14 (2.4)	0 (0)	Ref.			
Dermatology	17 (2.9)	0 (0)	0.000 (0.000–)	0.998		
Family medicine	9 (1.5)	0 (0)	0.000 (0.000–)	0.999		
Internal medicine	139 (23.5)	0 (0)	0.000 (0.000–)	0.995		
Laboratory medicine	29 (4.9)	0 (0)	0.000 (0.000–)	0.998		
Obstetrics and gynecology	45 (7.6)	0 (0)	0.000 (0.000–)	0.997		
Ophthalmology	56 (9.5)	0 (0)	0.000 (0.000–)	0.997		
Pediatric	46 (7.8)	0 (0)	0.000 (0.000–)	0.997		
Psychiatry	5 (0.8)	0 (0)	0.000 (0.000–)	0.999		
Surgery	73 (12.4)	0 (0)	0.000 (0.000–)	0.996		
Others	158 (26.7)	2 (1.3)	0.013 (0.003–0.052)	< 0.001		
**Experience of general practitioner**						
Less than 5 years	318 (71.3)	2 (0.6)	Ref.			
More than 5 years	128 (28.7)	0 (0)	0.000 (0.000–)	0.995		
**Attended national conference**						
No	767 (61)	1 (0.1)	Ref.		Ref.	
Yes	490 (39)	2 (0.4)	0.004 (0.001–0.016)	< 0.001	0.000 (0.000–)	1.000
**Attended local conference**						
No	836 (66.5)	1 (0.1)	Ref.		Ref.	
Yes	421 (33.5)	2 (0.5)	0.005 (0.001–0.019)	< 0.001	0.001 (0.000–)	1.000
**Attended international conference**						
No	1187 (94.4)	2 (0.2)	Ref.		Ref.	
Yes	70 (5.6)	1 (1.4)	0.014 (0.002–0.104)	< 0.001	606512615.87 (0.000–)	1.000
**Have you ever received information about monkeypox during your studies in medicine?**						
No	1179 (93.8)	2 (0.2)	Ref.		Ref.	
Yes	78 (6.2)	1 (1.3)	0.013 (0.002–0.093)	< 0.001	647078.34 (0.000–)	1.000
**Have you ever heard of monkeypox before?**						
No	557 (44.3)	1 (0.2)	Ref.		Ref.	
Yes	700 (55.7)	2 (0.3)	0.003 (0.001–0.011)	< 0.001	29876.89 (0.000–)	1.000
**When did you first hear about monkeypox?**						
I had never heard of it	64 (5.1)	1 (1.6)	Ref.		Ref.	
A few days or weeks ago	663 (52.7)	1 (0.2)	0.002 (0.000–0.011)	< 0.001	0.000 (0.000–)	1.000
A month or so ago	530 (42.2)	1 (0.2)	0.002 (0.000–0.013)	< 0.001	0.000 (0.025–)	1.000

OR, odds ratio; 95% CI, 95% confidence interval; Ref., reference value

Bold figures are significant values.

The majority (53.7%, *n* = 675) were medical students, followed by resident physicians (25.4%, *n* = 319), specialists (12.2%, *n* = 153), and general practitioners (8.8%, *n* = 110). Around 24.5% (*n* = 166) of medical students were in their fifth year of medical study, followed by sixth year (20.5%; *n* = 139), second year (19.8%; *n* = 134), fourth year (15.6%; *n* = 106), third year (10.6%; *n* = 72), and first year (9%; *n* = 61). With regard to medical speciality, 139 (23.5%) were internal medicine doctors, while 158 (26.7%) were specialists in areas not included in the questionnaire. 71.3% of general practitioners (*n* = 318) had less than 5 years’ experience. Around 39% of respondents (*n* = 430) had attended a national conference, 33.5% (*n* = 421) a local conference, and 5.6% (*n* = 70) an international conference. Only 6.2% (*n* = 78) of participants acquired knowledge about monkeypox throughout their medical education. Over half of the participants (55.7%, *n* = 700) had heard of monkeypox previously. Around 52.7% (*n* = 663) of respondents reported hearing about monkeypox for the first time a few days or weeks ago, whereas 42.2% (*n* = 530) heard about it a month ago.

#### Information sources

Table 2 shows the information sources used by healthcare workers (HCWs) for information on monkeypox. Most responders (89.6%) reported obtaining knowledge about monkeypox through social media. 75% of respondents used the internet for information about monkeypox. Fewer than half of respondents (46.4%) obtained knowledge from their friends, while 32.6% relied on television. Nearly 29.2% of respondents received information from health sector professionals. Only 8.6% of participants considered medical lectures as a source of knowledge about monkeypox. The respondents who obtained information from a medical seminar had the greatest percentage of excellent knowledge (0.9%), followed by those who acquired information from the internet, social media, friends, fellow healthcare workers, and television (Table 2).

**Table 2:** Sources of information on monkey virus, based on good knowledge score (using a cut-off of 70%) (*n* = 1257)

Sources of information received on virus	*n* (%)	Good knowledge, *n* (%)
Social media	1126 (89.6)	3 (0.3)
TV	410 (32.6)	1 (0.2)
Medical seminars	108 (8.6)	1 (0.9)
Friends	583 (46.4)	2 (0.3)
Health sector workers	367 (29.2)	1 (0.3)
Internet	949 (75.5)	3 (0.3)

### Knowledge and associated determinants

The mean and median values for knowledge were 10.23 and 11, respectively, out of a possible total of 34, with a range of 0–25. Using a cutoff score of 70%, 0.23% (3 of 1257) of individuals had correct knowledge. The majority of participants had a low degree of knowledge of monkeypox. Only 2.7% and 33.3% of respondents correctly recognized the monkeypox's animal host and incubation period, respectively. Around 39.9% of responders provided the proper transmission pathway for monkeypox. Only 1.8% of respondents correctly identified the signs and symptoms of monkeypox. Approximately 25.1% of respondents correctly recognized the duration of monkeypox disease.

Questions on the diagnostic methods used, sample types examined, treatment process, preventive measures, and signs of complications were correctly answered by 36.7%, 44.7%, 19.6%, 17.5%, and 0.6% of respondents, respectively. Approximately 23.9% of participants correctly identified the psychological impact of monkeypox. Nearly 12.6% of respondents correctly identified the monkeypox death rate. 74.2% correctly stated that monkeypox is caused by a virus. 30.5% of respondents felt that monkeypox is commonly spread from human to human, while 41.8% believed that it may be transmitted by an infected monkey's bite.

Some 24.1% of respondents indicated that American travelers were the primary source of imported monkeypox cases. More than half of respondents (60%) thought that the symptoms of monkeypox and smallpox are similar. However, other questions on symptoms and signs were answered correctly by only a minority of respondents. About 43.8% of respondents identified the usage of paracetamol as a potential management option for monkeypox. In addition to symptomatic therapy, 35.2% of respondents said that antiviral medication is required for the management of monkeypox, while 9.1% said that antibiotics are not necessary to treat human monkeypox (Supplementary Table 1).

The connection between the explanatory variables and knowledge (good versus poor) was examined using 70% of 34 questions as the threshold; no significant correlation was observed between any of the explanatory factors and knowledge. Nevertheless, a multivariate analysis revealed that socioeconomic status, general practitioner experience, attendance at a national conference, whether information about monkeypox was received during medical school, whether the individual had previously heard of monkeypox, and the time at which the individual first heard about monkeypox were strongly linked with knowledge level when adopting a lower threshold (score of 70%) (Table 1).

Table 1 also includes unadjusted and multivariable logistic regression analysis, showing predictors of knowledge about human monkeypox infection among healthcare professionals in the Syrian Arab Republic. No statistically significant associations were found between predictor variables and knowledge regarding monkeypox (*p* > 0.05) (Table 1).

## Discussion

Our study examined the level of MPXV awareness among Syrians with medical backgrounds. At first glance, it is evident from the collected data that Syrian medical groups possess severely inadequate MPXV knowledge. It is worth mentioning that Syria declared itself to be free of MPXV while our survey was taken [13]. Nevertheless, it is vital for emergency doctors and general practitioners to be able to properly detect, diagnose, and provide initial treatment for new possible MPXV cases. The relatively low incidence rate of the virus in Syria may be partially responsible for these low knowledge levels. Other possible factors include the relatively poor health education system and the worsening economic situation.[14]

Our findings were consistent with previous studies. A cross-sectional study of 606 healthcare workers conducted in one Middle Eastern country reported that only four out of the 11 knowledge questions relating to human monkeypox virus were answered correctly by more than 50% of respondents [15]. Another study conducted in Saudi Arabia among 314 medical students found that around 72% of participants had poor knowledge on monkeypox virus [16]. Similar findings were also seen among Jordanian healthcare students, with just three of 11 monkeypox questions being answered correctly by 70% of respondents [15]. Regarding acknowledgment of MPXV, only 55.7% of respondents were aware of the existence of MPXV. This is in contrast with a study conducted in Indonesia, in which 91.9% of respondents had heard of MPXV [12].

The vast majority of respondents denied studying MPXV as part of their education curriculum. Furthermore, formal medical plans are not being prepared for the worst-case epidemic scenario due to a lack unawareness of such a threat. As a result, it is crucial to include MPXV and other protentional pandemic-causing viruses in future educational programs. As shown in Table 1, all specialties demonstrated equally poor overall knowledge of MPXV. In contrast with the Indonesian study [12], our results showed that medical students demonstrated better knowledge levels than both general practitioners and residents. The same applied to younger participants, who had a greater chance of higher results than older respondents. As expected, the young are more accustomed to the internet, and hence have greater access to information, including reports on MPXV. Moreover, it is reasonable to suppose that older physicians are busier in their work and may have poorer technological abilities. Despite the poor overall knowledge shown by our study, Table 2 shows that social media and the internet were the primary information sources for respondents with the higher levels of expertise.

As a result of our findings, below are some suggestions for ways to improve knowledge of monkeypox among Syrian clinicians and undergraduate medical students:

○ Efforts should be made to enhance the roles of social media and the internet, particularly during pandemics, in order to improve information availability and prevention strategies [17], [18].
○ Knowledge gaps exist across different health professions, suggesting that sector-specific education initiatives are required. Given the low levels of knowledge reported among doctors and nurses in this studu, it stands to reason that increasing their knowledge of human monkeypox (HMPX) would have a positive effect on increasing their confidence in their ability to diagnose and treat the condition [19].
○ Public knowledge of monkeypox illness could be improved through lectures, seminars, and the use of electronic and print media to provide up-to-date information.
○ Earlier studies have reported that reducing the spread of false information during an epidemic has been proven to enhance public health outcomes. Social media facilitated the rapid spread of false information during the COVID-19 pandemic, which was later termed an ‘infodemic’. This phenomenon was evident during the multi-country HMPX epidemic occurring in 2022 [19]. Thus, the government must take measures to dispel any non-scientific myths arising during infectious disease outbreaks.
○ There must be an emphasis on the value of experts and scientists reporting promptly and accurately on the causes and locations of infectious disease epidemics.
○ The successful elimination of HMPX virus requires prompt patient recognition, the timely reporting of suspected cases to health authorities, and the implementation of public health intervention measures.
○ It is strongly recommended that the smallpox vaccination program be maintained and that more antiviral drugs are produced to tackle this mostly unnoticed tropical infection.

### Limitations

Our study showed some limitations. Due to the nature of our online survey and the requirement for internet connection, there was a potential bias in geographical selection, although efforts were made to distribute the survey as equally as possible. Participants may have searched for correct answers while completing the survey and caused a dishonesty bias. Nevertheless, it was clearly stated at the beginning of the survey that participants should only respond to questions based on current knowledge, without external aid.

## Conclusion

The Syrian clinicians and the undergraduate medical students in our study demonstrated an inadequate degree of understanding about monkeypox. Since these physicians need to be aware of this sickness, including its diagnosis, treatment, risk factors, and preventative measures, particularly because global health agencies have recognized monkeypox as being a pandemic of global concern, numerous practical recommendations for resolving this current issue have been presented in this study.

## Appendix Supplementary materials

Image, application 1
