# Supplementary material for: Monkeypox in Syria: Highlighting an awareness issue
Source: IJID Reg. 2023 Apr 20;7:271–6. doi: 10.1016/j.ijregi.2023.04.008 (PMC10116148; doi:10.1016/j.ijregi.2023.04.008)
Supplement: Supplementary file 1 [file mmc1.docx]

**Supplementary Table 1.** Assessment of monkeypox knowledge among healthcare professionals in Syria (*n* = 1257)

| **Knowledge assessment** | **Correct answer, *n* (%)** |
| --- | --- |
| 1. The natural host for this virus (Squirrels, Cows, Dog, Rats, Monkeys, I don't know, None of the above) | 27 (2.7) |
| 2. The incubation period for the virus (1–7 days, 7–14 days, 24–28 days, I don't know, None of the above) | 418 (33.3) |
| 3. The age group most at risk of infection (over 50 years old, 40–50 years old, under 40 years old, It affects all ages the same, I don't know, None of the above) | 155 (12.3) |
| 4. Infection is transmitted through (From animal to human, Breathing path, From pregnant mother to fetus, Fecal–oral route, Through body secretions, Common items (clothing), I don't know, None of the above) | 501 (39.9) |
| 5. Signs and symptoms of monkeypox (Fever, Headache, Muscle pain, Cough, Vomiting, Swollen lymph nodes, Exhaustion, Rash, I don't know, None of the above) | 23 (1.8) |
| 6. Duration of illness due to monkeypox (1 week, 2–4 weeks, 4–6 weeks, 6–8 weeks, I don't know, None of the above) | 316 (25.1) |
| 7. Diagnostic tools for monkeypox (Clinical diagnosis, PCR, Microscope, Culture and sensitivity, Sera, I don't know, None of the above) | 461 (36.7) |
| 8. Examination sample type (Stool, Urine, Skin lesion fluid, Blood, I don't know, None of the above) | 562 (44.7) |
| 9.Treatment (Supportive (fluids and nutrients), Soothing, Antibiotics, Antivirals, Antimalarials, Antifungals, I don't know, None of the above) | 246 (19.6) |
| 10. Prevention (Good hand hygiene, Use of personal protective equipment, Isolate infected patients from others at risk, Vaccination, Avoiding contact with animals that may harbor the virus, I do not know, None of the above) | 220 (17.5) |
| 11. Complications (Skin complications, Ocular complications, Respiratory complications, Central nervous system complications, Dehydration (vomiting, diarrhoea, poor eating), Sepsis, Urinary tract infection, Growth retardation, death, I don't know, None of the above) | 8 (0.6) |
| 12. Psychological effect (Depression, Stigma, Psychosis, Suicidal thoughts, I don't know, None of the above) | 300 (23.9) |
| 13. Mortality rate (10–15%, 1–10%, Less than 1%, 15–20%, More than 20%, I don't know) | 158 (12.6) |
| 14. Monkeypox is prevalent in Southeast Asian countries (Yes/No/Don't know) | 224 (17.8) |
| 15. Monkeypox is prevalent in Western and Central Africa (Yes/No/Don't know) | 624 (49.6) |
| 16. There have been many human monkeypox cases in the Arab world (Yes/No/Don't know) | 321 (25.5) |
| 17. There is an outbreak of human monkeypox in the Arab world (Yes/No/Don't know) | 53 (4.2) |
| 18. Monkeypox is a viral disease infection (Yes/No/Don't know) | 933 (74.2) |
| 19. Monkeypox is a bacterial disease infection (Yes/No/Don't know) | 34 (2.7) |
| 20. Monkeypox is easily transmitted human-to-human (Yes/No/Don't know) | 383 (30.5) |
| 21. Monkeypox could be transmitted through a bite from an infected monkey (Yes/No/Don't know) | 525 (41.8) |
| 22. Travelers from the American continent are the main source of imported cases of monkeypox (Yes/No/Don't know) | 303 (24.1) |
| 23. Monkeypox and smallpox have similar signs and symptoms (Yes/No/Don't know) | 754 (60) |
| 24. Monkeypox and smallpox have the same signs and symptoms (Yes/No/Don't know) | 308 (24.5) |
| 25. A flu-like syndrome is one of the early signs or symptoms of human monkeypox (Yes/No/Don't know) | 453 (36) |
| 26. Rashes on the skin are one of the signs or symptoms of human monkeypox (Yes/No/Don't know) | 823 (65.5) |
| 27. Papules on the skin are one of the signs or symptoms of human monkeypox (Yes/No/Don't know) | 583 (46.4) |
| 28. Vesicles on the skin are one of the signs or symptoms of human monkeypox (Yes/No/Don't know) | 649 (51.6) |
| 29. Pustules on the skin are one of the signs or symptoms of human monkeypox (Yes/No/Don't know) | 647 (51.5) |
| 30. Lymphadenopathy (swollen lymph nodes) is one clinical sign or symptom that could be used to differentiate monkeypox and smallpox cases (Yes/No/Don't know) | 540 (43) |
| 31. One management option for symptomatic monkeypox patients is to use paracetamol (Yes/No/Don't know) | 550 (43.8) |
| 32. Antivirals are required in the management of human monkeypox patients (Yes/No/Don't know) | 442 (35.2) |
| 33. Antibiotics are required in the management of human monkeypox patients (Yes/No/Don't know) | 114 (9.1) |
| 34. Diarrhoea is one of the signs or symptoms of human monkeypox (Yes/No/Don't know) | 206 (16.4) |
